# Cancer megafunds with *in silico* and *in vitro* validation: accelerating cancer drug discovery *via* financial engineering without financial crisis

**DOI:** 10.18632/oncotarget.9808

**Published:** 2016-06-03

**Authors:** Xianjin Yang, Edouard Debonneuil, Alex Zhavoronkov, Bud Mishra

**Affiliations:** ^1^ King Abdullah University of Science and Technology, Jeddah, Saudi Arabia; ^2^ Universite Lyon 1, Laboratoire SAF, France; ^3^ Insilico Medicine, ETC, Johns Hopkins University, Baltimore, MD, USA; ^4^ Courant Institute and Tandon School of Engineering, NYU, NY, USA

**Keywords:** megafund, in silico validation, cancer megafund, research-backed obligation, RBO

## Abstract

Advances in financial engineering are radically reshaping the biomedical marketplace. For instance, new methods of pooling diversified drug development programs by placing them in a special purpose vehicle (SPV) have been proposed to create a securitized cancer megafund allowing for debt and equity participation. In this study, we perform theoretical and numerical simulations that highlight the role of empirical validation of the projects comprising a cancer megafund. We quantify the degree to which the deliberately designed structure of derivatives and investments is key to its liquidity. Research megafunds with comprehensive *in silico* and laboratory validation protocols and ability to issue both debt, and equity as well as hybrid financial products may enable conservative investors including pension funds and sovereign government funds to profit from unique securitization opportunities. Thus, while hedging investor's longevity risk, such well-validated megafunds will contribute to health, wellbeing and longevity of the global population.

## INTRODUCTION

Biomedicine faces a dilemma. Despite many recent scientific breakthroughs demonstrating a clear potential for combating cancer, there has been no significant private investment in cancer drug R&D. Both constantly rising costs and increasing rates of failures in the late stages of clinical trials have made the pharmaceutical R&D unappetizingly risky from a financial perspective [1].

In particular, there are two main challenges. First, on average the success rate of clinical trials is low so that the average financial yield is low. Second, the large investments required to bring a single treatment to the market lead to an all-or-nothing result: the risk is high. To increase funding for cancer research while providing adequate financial returns to investors with wide ranging risk profiles by investing in multiple clinical trials at once thereby mutualizing investments and diluting risks, the concept of a “cancer megafund” was proposed [2]. A massive amount of investment capital would support a portfolio of many drug development projects in order to spread the risks associated with any stand-alone biomedical project. The resulting lowered default probabilities could make returns attractive to investors. By issuing Research-backed Obligations (RBOs), it could be also possible to attract both fixed-income and equity investors.

In parallel, a comprehensive multi-period, multi-state program was developed to simulate the behavior of the megafund entity over time and stress test the conceptual framework. Fagnan et al. [3] extended it in a way that demonstrated that third-party guarantees can improve the economics of RBO transactions at very low costs. The megafund concept was then extended to orphan diseases [4] and to longevity hedge instruments [5]. The approach received criticism with calls made for more mathematically rigorous and faithful modeling, which could result in structuring and simulating the megafund entities to better elucidate and engineer risk profiles [6].

One alternative is to group investments to attract a diverse investor base. The original cancer megafund concept proposed offloading assets into one (SPV), a commonly used type of legal structure to make the link between investors and the users of investments such as pharmaceutical companies, without considering the heterogeneity of the drug development programs. In practice, drug development programs may typically be housed under different SPVs to attract diverse investors. For example, some investors may prefer investing in immunotherapy and others may prefer investing in small molecules tethered to nanoparticles. Another major challenge of operating in the real world is the presence of “lemons” [7] in drug development programs, where projects have flaws known to their promoters but not to the buyers which can be modeled as information asymmetric games with potential for deceptive Nash equilibria. It is suspected that drug discovery and development is a “lemons” market, where over half of results may be non-reproducible in part due to the complexity of experimental conditions, the pressure to publish, low statistical powers and difficulty to publish negative results [8]. Therefore, while there are many efforts to consolidate knowledge [9], it might be expected that lemons are a practical important factor to consider when devising a megafund.

Through stylized examples, this study demonstrates how the introduction of “lemons” and their distributions influence the profits gained from different tranches: an “ideal” megafund discarding lemons before they are included into SPVs, a “reliable” megafund distributing the lemons evenly in the megafund and an “unfair” megafund greedily maximizing short term profit by placing lemons into toxic SPVs. Because proposed drug development projects are not a typical financial asset, we demonstrate that careful “validation” of their quality can in the best case lead to a better selection of what programs to develop and in the worst case lead an unfair megafund to better create toxic SPVs.

Our stylized “ideal” megafund does not fund any lemon at all. This is a hypothetical fund utilizing rigorous validation mechanisms and enough substantial time and resources to scrutinize every project before including it into an SPV. Table 1 shows that an ideal megafund has very low risks and very high financial returns and that making several SPVs of about 50 drug development programs enjoys many attractive traits. In addition to attracting different types of investors, it is optimal from the financial perspective.

**Table 1:** Summary statistics of different biomedical megafunds

“150 assets, serving 8.5% to senior tranches	Senior tranches in practice		Equity tranches in practice	
	Yield	Default Probability	Yield	Default Probability
Ideal megafund, 1 SPV Ideal megafund, 6 SPVs	8.5% 8.45%	< 0.1% 0.4%	27.1% 27.2%	< 0.1% < 0.1%
Reliable megafund, 1 SPV Reliable megafund, 6 SPVs	8.44% 7.78%	1.6% 6.5%	17.2% 17.5%	5.0% 5.0%
Unfair megafund, 6 SPVs	5.85%	19.9%	27.7%	5.0%
**“150 assets, serving 8.5% to senior tranches**	**Senior tranches in practice**		**Equity tranches in practice**	
	**Yield**	**Default Probability**	**Yield**	**Default Probability**
Ideal megafund, 1 SPV Ideal megafund, 6 SPVs	16.8% 16.6%	0.2% 2.8%	24.0% 24.1%	0.6% 0.6%
Reliable megafund, 1 SPV Reliable megafund, 6 SPVs	14.8% 14.8%	22.7% 25.1%	12.2% 12.2%	36.7% 36.7%
Unfair megafund, 6 SPVs	11.7%	41.8%	25.9%	36.7%

Our stylized “reliable” megafund does not perform extensive due diligence like the ideal megafund but will randomly choose “lemons” alongside other projects and distribute them evenly among SPVs. The case of a single SPV then corresponds to the case studied by Fernandez et al. [2] and indeed Table 1 shows that low risks and potentially sufficient returns can be obtained when pooling 150 assets. Making several SPVs to attract diverse investors generates non negligible risks in senior tranches, thus making one naturally wonder if returns can be high enough to attract investors.

Our stylized “unfair” megafund shows that the situation can rapidly deteriorate with multiple SPVs as after diligent examinations the managers of the megafund may identify the set of lemons and be incentivized to not pool assets completely randomly. As indicated by Sanjeev Arora et al. [10], a strategy is to over represent the number of lemon projects in a few SPVs, thereby skewing the probability of default while making it computationally intractable to detect the toxic SPVs. Other SPVs would be handled in the same way as with the reliable megafund. Table 1 shows that equity tranches would then perform better than in the reliable megafund but that senior tranches would massively default. If the megafund managers hold shares of equity tranches, as generally done for the sake of credibility and responsibility, when facing lemons they would actually be tempted to manage the fund in an unfair way.

Average annual yield and probability of loss of senior and equity tranches, for a megafund of 150 drug development programs that aims to serve a respectable return to senior tranches, strongly depend on the type of megafund. These statistics suggest using validation mechanisms to get closer to an ideal megafund. The underlying mathematics and parameters are in the next section.

The results in Table 1 clearly indicate that it would be ideal to eliminate lemons beforehand. Such a validation-based strategy may actually be feasible at a certain cost: initial *in vitro*, *in vivo* and *in silico* intense “validation” could filter some lemons out. It could also improve the knowledge on how to develop non-lemons: what population to target, the way of administration and the dosage all jointly maximizing some measures of benefit/risk. It can therefore be expected that investing in such validation methods can greatly improve the performance of megafunds.

**Figure 1:**
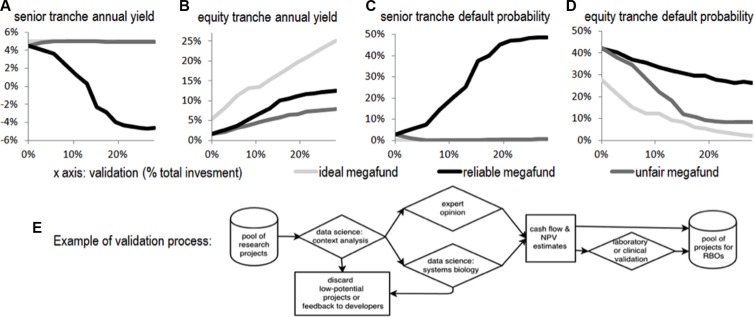
The relations between average yields and the degree of validation

In order to investigate such aspects, we used the simulation framework of Fernandez et al. [2] and its extension by Fagnan et al. [3]. We further extended it to model multiple SPVs, to distinguish lemons from non-lemons, to model the three behaviors of megafunds described above and to model the impact of initially investing in validation. The results are in Figure 1.

Figure 1 shows the average yields (A and B) and default probabilities (C and D) for respectively senior and equity tranches over 20,000 simulated paths for an ideal megafund (light gray lines), a reliable megafund (dark gray lines) and an unfair megafund, depending on the degree of validation (as a percentage of the total investment; x-axis). As expected, validation reduces risks and improves returns for ideal and reliable megafunds; here, it turns some poorly attractive fund into strongly attractive funds. In case of an unfair management however, the knowledge of where lemons are lead the fund managers to collect them into toxic SPVs, which makes the corresponding senior tranches very risky. Using a portion of the investments for a validation process that could for example be strongly data driven (E) may significantly improve the performance of the fund by reducing the number of lemons.

To allow readers to analyze their own preferred parameters, we uploaded the simulation software in the public domain with an open-source license to run, modify and distribute the code and provided the mathematical model in the next section. One can choose higher returns for senior tranches or otherwise adjust other parameters or mechanisms. Figure 1 however already points to the main conclusion of the paper: the behavior of a cancer megafund strongly depends on the science behind its assets and on the transparency about lemons; treating lemons flippantly can be catastrophic.

As for Akerlof's original model of used car markets [7] or even the 2008 credit crunch crisis, knowing the quality of assets and behaving in an informed manner with respect to bad assets are crucial practical aspects of a megafund. As a market of megafunds emerges, investors, lacking means to distinguish the type of a megafund [10], may choose either not to participate or to demand lower prices. Similarly to the “lemon” market for used automobiles, ideal and reliable megafunds that perform costly investigations will then be expelled from the market by low cost deceptive megafunds that fund deceptive biomedical research.

## MATHEMATICAL APPROACH

### Overview

We start by modelling a reliable megafund with several SPVs. A series of stylized assumptions are taken for simplicity. Examples of parameters are given for clarity.

Having a single SPV (*M = 1*) is then simply a special, but particularly interesting case. An ideal megafund (no lemon: *n* = 0) is another interesting special case.

The unfair megafund can be viewed as a combination of two reliable megafunds for the senior tranches and as a reliable fund with slight adaptations for the equity tranches.

### Modelling a reliable megafund

#### Drug development programs, lemons and SPVs

As an example, out of 300 investigated drug development programs a reliable megafund selects *N* = 150 of them, and distributes them uniformly across *M* = 10 SPVs of *D* = 50 drug development programs each. Let us consider for simplicity that each drug development program is shared across the same number of SPVs, which is then *M D/N*.

Out of all, we assume that *n* = 75 drug development programs are in fact lemons and that for simplicity their success rate is 0. The other half is non-lemons, their success rate is *p* = 10% (so that the combined average success rate is 5% as in [2]). In case a non-lemon succeeds it generates *B* = 12.3 millions of present value at *T* = 10 years (for simplicity). We assume that the upfront investment is *I* = 0.2 million for every drug development project, whether a lemon or not.

#### Senior tranche and equity tranche

In order to attract both fixed-income and equity investors, *s* = 50% of the investment capital of any SPV goes to a senior tranche and the remaining part (1 − *s* = 50%) to an equity tranche. For simplicity, each SPV contains two tranches. After ten years, the senior tranche pays the gains of the SPV up to the initial investment plus an interest that corresponds to an *r* = 5% annual interest rate. We say that the senior tranche “defaults” if it is not able to pay that interest rate.

The remaining gains are spread to the equity tranches of all SPVs as if it was a unique equity tranche (method that reduces the risk of equities). The equity amount is then paid to its investors. If it is below the capital invested in the equity tranche we say that the latter “defaults”.

### Computing financial characteristics of a reliable megafund

#### Payoff of senior tranches

The total investment in the megafund is *NI*: the product of the number investments *N* and the amount invested in each *I*. It is split across SPVs and across tranches so the investment for a senior tranch is $I_{\text{senior}}=\frac{\text{NI}}{M}s$. The senior tranche pays *I*_*senior*_ (1 + *r*)^*T*^ to its investors if there are enough successes in the SPV to do so. If there are not enough successes, i.e. if the senior tranche defaults, the few successes each pay $\frac{\text{BN}}{\text{MD}}$ to the investors (because as seen above each drug development program is split across *MD/N* SPVs).

#### Default of senior tranches

The frontier between the two cases determines the probability of default *d*_*senior*_ : when k successes that pay as much as what the senior tranche shall pay in the absence of default i.e. $k\frac{\text{BN}}{\text{MD}}=\left(\text{NIs}/M\right){\left(1+r\right)}^{T}$. The number of successes must be an integer so we round *k* up to the nearest integer above K=⌈k⌉ and the senior tranch defaults if the number of successes is less than

$$K=\left\lceil \left(\text{NIs}/M\right){\left(1+r\right)}^{T}\frac{\text{MD}}{\text{BN}}\right\rceil =\left\lceil \text{DIs}/B{\left(1+r\right)}^{T}\right\rceil .$$

The default probability of the senior tranche is then

$$d_{\text{senior}}=\sum_{k=0}^{K-1}\left(\begin{array}{c} \text{NL}\\ k \end{array}\right)p^{k}{\left(1-p\right)}^{\left(\text{NL}-k\right)},$$

where $\text{NL}=D\frac{N-n}{N}$ is the number of non lemons in the SPV.

#### Yield of senior tranches

The average value generated by the senior tranche is the average of what it pays weighted by its probability:

$$\begin{array}{ll} V_{\text{senior}} & =\left(1-d_{\text{senior}}\right)I_{\text{senior}}{\left(1+r\right)}^{T}\\ & +\sum_{k=0}^{K-1}\left(\begin{array}{c} \text{NL}\\ k \end{array}\right)p^{k}{\left(1-p\right)}^{\left(\text{NL}-k\right)}k\frac{\text{BN}}{\text{MD}}. \end{array}$$

The average annual yield is then expressed from the investment and the average return:

$$y_{\text{senior}}={\left(\frac{V_{\text{senior}}}{I_{\text{senior}}}\right)}^{\frac{1}{T}}-1.$$

#### Yield of equity tranches

All equity tranches receive the same investment $I_{\text{equity}}=\frac{\text{NI}}{M}\left(1-s\right)$. Also, all equity tranches receive the same values: for every SPV, the values in excess of a senior tranche are spread over all equity tranches. So all equity tranches are in the same state and it is easier to consider them together as one large equity tranche that receives the known amount I_equities_ = *NI*(1 − *s*) and pays the value of all successes in the megafund minus the payments of all the senior tranches. On average the megafund has (*N* − *n*)*p* successes so that aggregate equity tranche pays

$$V_{\text{equities}}=\left(N-n\right)\text{pB}-{\text{MV}}_{\text{senior}}.$$

The average annual yield is then

$$y_{\text{equity}}={\left(\frac{V_{\text{equities}}}{I_{\text{equities}}}\right)}^{\frac{1}{T}}-1.$$

#### Default of equity tranches

That calculation is complicated in the general case so we performed case by case calculations.

The calculation is simple in the case of a megafund with a single SPV: the reasoning is that of *d_senior_* with a *K* that is the minimal number of successes to pay *P*_*senior*_ + *I*_*equity*_:

$$d_{\text{quity}}^{M=1}=\sum_{k=0}^{K'-1}\left(\begin{array}{c} N-n\\ k \end{array}\right)p^{k}{\left(1-p\right)}^{\left(N-n-k\right)},$$

where

$$K'=\left\lceil \frac{\left(\text{NIs}\right){\left(1+r\right)}^{T}+\text{NI}\left(1-s\right)}{B}\right\rceil .$$

In the case of multiple SPVs, a long formula could be established for the equity default probability by considering the two-dimensional enumeration of how many senior tranches default and how many excess successes are generated in the group of SPVs whose senior tranches do not default. We used an intuitive approximation instead.

A number *k* of successes occur within the whole megafund and generate a value *kB*. On average *MV*_*senior*_ must be subtracted from that value in order to consider how much value remains for equity – that is where the approximation is done: we consider *MV*_*senior*_ as given whereas it is a random variable. The equity tranches default if that remaining value is insufficient to pay the equity investments i.e. if *kB* − *MV*_*senior*_ < *I*_*equities*_. So the default is when *k* < *K*' where

$$K'\approx \left\lceil \frac{{\text{MV}}_{\text{senior}}+\text{NI}\left(1-s\right)}{B}\right\rceil .$$

That is,

$$d_{\text{equity}}=\sum_{k=0}^{K'-1}\left(\begin{array}{c} N-n\\ k \end{array}\right)p^{k}{\left(1-p\right)}^{\left(N-n-k\right)}.$$

We will use a more intuitive estimation of *K*′:

$$K'\approx \left\lceil \frac{M\left(\text{NIs}\right){\left(1+r\right)}^{T}+\text{NI}\left(1-s\right)}{B}\right\rceil .$$

Indeed it is a simple formula that matches the exact formula in the case of a single SPV and that is otherwise numerically close to the formula above and comparatively prudent.

#### Computing financial characteristics of single SPV and ideal megafunds

The analysis for single SPV megafunds can then be slightly simplified by setting *M* = 1 and *D* = *N* in the above equations. The equity default probability in particular is simple as indicated in the previous section.

### Modelling unfair megafunds and computing associated characteristics

#### Modelling

The *N* assets are now split in two types of SPVs: *M*_1_ SPVs have *d*_1_ lemons each and *M*_2_ toxic SPVs have *d*_2_ lemons each with *d*_2_ > *d*_1_.

#### Senior default probability

It is as if we have two reliable megafunds: a non-toxic megafund with $N_{1}=N\frac{M_{1}}{M_{1}+M_{2}}$ assets and *M*_1_ SPVs and a toxic megafund with *N*_2_ = *N* − *N*_1_ assets and *M*_2_ SPVs.

Each of them has a default probability

$$d_{i,\text{senior}}=\sum_{k=0}^{K-1}\left(\begin{array}{c} D-d_{i}\\ k \end{array}\right)p^{k}{\left(1-p\right)}^{\left(D-d_{i}-k\right),}$$

where *i* = 1, 2 and $K=\left\lceil \text{DIs}/B{\left(1+r\right)}^{T}\right\rceil$ is the same for the two megafunds. The overall default probability is then of course the weighted average

$$d_{\text{senior}}=\frac{M_{1}}{M}d_{1,\text{senior}}+\frac{M_{2}}{M}d_{2,\text{senior}}.$$

#### Senior yield

Similarly

$$y_{\text{senior}}={\left(\frac{M_{1}V_{1,\text{senior}}+M_{2}V_{2,\text{senior}}}{MI_{\text{senior}}}\right)}^{\frac{1}{T}}-1,$$

where

Vi,senior=(1−di,senior)Isenior(1+r)T+∑k=0K−1(D−dik)pk(1−p)(D−di−k)kBNMD.

#### Equity yield

The same reasoning as for a reliable megafund leads exactly to the same formulas.

#### Equity default probability

A long formula could be established by considering the three-dimensional enumeration of how many senior tranches default in the two sub-megafunds and how many excess successes are generated in the group of SPVs whose senior tranches do not default. We instead use the approximation concept used for a reliable megafund, it leads to the same formula.

#### Numerical application

The following Table 2 is a detailed version of Table 1 presented earlier in the paper. In all cases, *N* = 150, *I* = 0.2, *B* = 13.6 and *s* = 50%.

**Table 2:** Detailed summary statistics under different configurations

Reliable 1	‘a la Fernandez et al.’	Reliable 2	‘a la Fernandez et al.’
Parameters	n=0, p=5%, M=1, r=3.8%	Parameters	n=0, p=5%, M=1, r=5%
Senior	K=⌈1.8⌉, d=0.4%, y=3.8%	Senior	K=⌈1.99⌉, d=0.4%, y=5.0%
Equity	$y=19.0\%,\;K^{'}=\left\lceil 2.99\right\rceil ,\;d=1.8\%$	Equity	$y=18.6\%,\;K^{'}=\left\lceil 3.2\right\rceil ,\;d=5.5\%$
Reliable 3	‘a la Fernandez et al.’	Reliable 4	‘a la Fernandez et al.’ on
Parameters	n=0, p=5%, M=1, r=8.5%	Parameters	n=0, p=5%, M=1, r=9.4%
Senior	K=⌈2.8⌉, d=1.8%, y=8.3%	Senior	K=⌈2.99⌉, d=1.8%, y=9.3%
Equity	$y=17.3\%,\;K^{'}=\left\lceil 3.98\right\rceil ,\;d=5.5\%$	Equity	$y=16.8\%,\;K^{'}=\left\lceil 4.2\right\rceil ,\;d=12.6\%$
Reliable 5	Reliable 3 lemons/non-lemons	Reliable 6	5 or 50 SPVs
Parameters	n=75, p=10%, M=1, r=8.5%	Parameters	n=75, p=10%, M>1, r=8.5% D=52 (previously N=D=150)
Senior	K=⌈2.8⌉, d=1.6%, y=8.4%	Senior	K=⌈0.96⌉, d=6.5%, y=7.8%
Equity	$y=17.2\%,\;K^{'}=\left\lceil 3.98\right\rceil ,\;d=5.0\%$	Equity	y=17.5%, K′=⌈3.98⌉, d=5.0%
Reliable 7	5 or 50 SPVs	Idea 1	1 SPV
Parameters	n=75, p=10%, M>1, r=16.8%	Parameters	n=0, p=10%, M=1, r=16.8%
Senior	K=⌈1.99⌉, d=25.1%, y=14.8%	Senior	K=⌈5.8⌉, d=0.2%, y=16.8%
Equity	$y=12.2\%,\;K^{'}=\left\lceil 6.98\right\rceil ,\;d=36.7\%$	Equity	y=24.0%,K′=⌈6.98,d⌉=0.6%
Ideal 2	5 or 50 SPVs	Ideal 3	
Parameters	n=0, p=10%, M>1, r=8.6% D=52	Parameters	n=0,p=10%,M>1,r=16.8% D=52
Senior	K=⌈0.96⌉, d=0.4%, y=8.5%	Senior	K=⌈1.99⌉, d=2.8%,y=16.6%
Equity	$y=27.2\%,\;K^{'}=\left\lceil 3.98\right\rceil ,\;d\leq 0.1\%$	Equity	$y=24.0\%,K^{'}=\left\lceil 6.98\right\rceil ,\;d=0.6\%$
Unfair		Unfair	
Parameters	n=75, p=10%, D=52, r=8.6% $M_{1}=M_{2}=3,\;d_{1}=9,\;d_{2}=43$	Parameters	n=75, p=10%, D=52, r=16.8% $M_{1}=M_{2}=3,\;d_{1}=9,\;d_{2}=43$
Senior	K=⌈0.96⌉, d=19.9%, y=5.9%	Senior	K=⌈1.99⌉, d=41.8%, y=11.7%
Equity	$y=27.6\%,\;K^{'}=\left\lceil 3.98\right\rceil ,\;d=5.0\%$	Equity	$y=25.9\%,\;K^{'}=\left\lceil 6.98\right\rceil ,\;d=36.7\%$

## SIMULATIONS

As with the work of Fagnan et al. [3], we focused only on early-stage investment (Preclinical and Phase I), which is the most risky portion of the drug development process and where funding is scarce. We selected one semester as the time step of the study and 6 years as the duration of the drug development process. During one semester, drugs have probabilities to move to another stage of the drug development process. At the end of each semester, current cash reserves will increase through the compound interest transferring to the next stage. We used the same methods as those used by Fernandez to make upfront payments and periodic payments and also to compensate the developers for successful completion of key milestones. If a drug successfully transfers into Phase II or other later stages, we sell it and realize profits immediately. For every drug, we used the same methods and parameters to evaluate it and calculate its cost as in the previous work.

It should be noted at this point that lemons have a different transition probability matrix from non-lemons. For non-lemons we use the same matrix as Fernandez et al. [2] as a reasonable assumption in the absence of validation (impacts of validation are described below). We designed the transition probability matrix of lemons based on the principle that lemons have much higher probability of failures and that the more stages they reach, the higher is the probability and cost of failure.

We introduce multiple SPVs and the behavior of the megafund: ideal, reliable or unfair. By default we start with 200 assets and consider that half of them are lemons. The ideal megafund starts by eliminating the lemons and therefore starts with *N* = 100. The reliable fund eliminates some of the lemons depending on the amount invested in validation and therefore starts with *N* between 100 and 200. The unfair fund keeps the *N* = 200 assets; in the absence of validation it behaves like the reliable fund (not able to distinguish between lemons and non-lemons) but the more the validation, the more it distributes lemons into some SPVs, the toxic SPVs. *M* = *N*/4 SPVs are built. Each SPV randomly chooses *D* = *N*/2 assets, where some asset will be distributed across a few SPVs, others across many SPVs: the only constraint is that each asset goes to at least one SPV. The unfair megafund uses a quarter of its SPVs as toxic SPVs.

The probability to detect that an asset is a lemon is modelled depending on the percentage of investments used for validation: *p*_*detection*_ = 1 − *e*^−10*v*^. With that formula it is 0 in the absence of validation and close to 95% when 30% of investments go to validation.

With validation also comes improvements of non-lemon probabilities. Indeed performing preliminary analysis on drugs should allow to better target a dose, way of administration and target population with a high benefit to risk balance.

The mechanism of tranches is minimally modelled for the sake of clarity of analysis. No junior tranche is used: we only model the senior tranche and the equity tranche. In case a senior tranche does not default it pays at the end of the period and the senior tranche pays nothing if it defaults.

## CONCLUSIONS

In conclusion, the megafund concept based on modern securitization techniques and debt and equity financing may provide another mechanism to accelerate drug discovery in cancer and other diseases. However, for the concept to be effective, it needs to consider the economics of the lemons market in cancer research. Considering the amount of irreproducible research published in high-level journals, it is fair to assume that asymmetry of information will exist between scientists, managers and investors. Introducing stringent *in silico* and laboratory validation techniques prior to enrolling projects into SPVs may improve the odds of clinical trials. A potential security mechanism would be that regulators impose that a significant percentage of the upfront costs go to drug discovery program validation and that results of investigations are openly shared with both debt and equity investors – this might also improve the reproducibility of biomedical research.
